# Ti Addition Effect on the Grain Structure Evolution and Thermoelectric Transport Properties of Hf_0.5_Zr_0.5_NiSn_0.98_Sb_0.02_ Half-Heusler Alloy

**DOI:** 10.3390/ma14144029

**Published:** 2021-07-19

**Authors:** Junsang Cho, Taegyu Park, Ki Wook Bae, Hyun-Sik Kim, Soon-Mok Choi, Sang-il Kim, Sung Wng Kim

**Affiliations:** 1 Department of Artificial Intelligence, Sungkyunkwan University, Suwon 16419, Korea; chojs@cyautotech.com; 2 Materials and Components Policy Division, Ministry of Trade, Industry and Energy, Sejong 30118, Korea; magnus76@hanmail.net; 3 R&D Center, CY Autotech Co., Ltd., Hwaseong 18336, Korea; baekw1991@gmail.com; 4 Department of Materials Science and Engineering, Hongik University, Seoul 04066, Korea; hyunsik.kim@hongik.ac.kr; 5 School of Energy, Materials and Chemical Engineering, Korea University of Technology and Education, Cheonan 31253, Korea; smchoi@koreatech.ac.kr; 6 Department of Materials Science and Engineering, University of Seoul, Seoul 02504, Korea; 7 Department of Energy Science, Sungkyunkwan University, Suwon 16419, Korea

**Keywords:** point defect, thermoelectric, half-Heusler, lattice thermal conductivity, phonon scattering

## Abstract

Compositional tuning is one of the important approaches to enhance the electronic and thermal transport properties of thermoelectric materials since it can generate point defects as well as control the phase evolution behavior. Herein, we investigated the Ti addition effect on the grain growth during melt spinning and thermoelectric transport properties of Hf_0__.5_Zr_0.5_NiSn_0.98_Sb_0.02_ half-Heusler compound. The characteristic grain size of melt-spun ribbons was reduced by Ti addition, and very low lattice thermal conductivity lower than 0.27 W m^−1^ K^−1^ was obtained within the whole measured temperature range (300–800 K) due to the intensified point defect (substituted Ti) and grain boundary (reduced grain size) phonon scattering. Due to this synergetic effect on the thermal transport properties, a maximum thermoelectric figure of merit, *zT*, of 0.47 was obtained at 800 K in (Hf_0__.5_Zr_0.5_)_0.8_Ti_0.2_NiSn_0.98_Sb_0.02_.

## 1. Introduction

The development of high-performance materials is a central issue in thermoelectrics since the conversion efficiency of thermoelectric (TE) system (both cooling and power generation systems as expressed in Equations (1) and (2) [1]) is directly determined by a dimensionless figure of merit (*zT* = *S*^2^*σT*/*κ*, where *S*, *σ*, *T*, and *κ* (or *κ*_tot_) are the Seebeck coefficient, the electrical conductivity, the absolute temperature, and the total thermal conductivity, respectively) of TE materials. The optimum coefficient of performance (*ϕ*_opt_) of a TE cooling system is given by

(1) $$\phi_{\mathrm{opt}}=\frac{T_{c}}{\Delta T}\frac{\sqrt{1+ZT_{m}}-T_{h}/T_{c}}{\sqrt{1+ZT_{m}}+1}$$

where *T*_c_ is the cold side temperature, *T*_h_ is the hot side temperature, Δ*T* = *T*_h_ − *T*_c_, *T*_m_ = (*T*_h_ + *T*_c_)/2, and *ZT*_m_ (TE figure of merit for a device) = (*z*_p_*T* + *z*_n_*T*)/2 (*z*_p_*T* and *z*_n_*T* are the *zT* of *p*-type and *n*-type TE materials), respectively. The theoretical maximum TE power generation efficiency (*η*_max_) is given by Equation (2).

(2) $$\eta_{\max}=\frac{\Delta T}{T_{h}}\frac{\sqrt{1+ZT_{m}}-1}{\sqrt{1+ZT_{m}}+T_{c}/T_{h}}$$

Among TE materials, the half-Heusler (HH) compound is one of the most important materials for mid- to high-temperature power generation applications due to their high thermal and mechanical reliability as well as high *zT* [2,3,4]. *zT* over 1.0 have been reported both in *n*-type HfNiSn-based and *p*-type NbFeSb-based HH alloys via the compositional tuning and nanostructuring approaches [5,6]; however, a long annealing process is required to obtain a complete single phase, which can show high *zT* and long-time stability. Due to the complexity in phase formation behavior according to temperature, suppression of the generation of full-Heusler and binary alloys is difficult in a conventional melt–solidification process [7]. In the previous study, we demonstrated that the complete single phase of *n*-type Ti_1−*x*_Hf*_x_*NiSn_1−*y*_Sb*_y_* half-Heusler alloys could be obtained via temperature-regulated melt spinning (TRMS, lower processing temperature than the required temperature to form full-Heusler phase) and spark plasma sintering (SPS) without any additional post-heat treatment [8,9]. Very recently, a single phase of a half-Heusler compound with a more complex composition of Hf_0.35_Zr_0.35_Ti_0.3_NiSn_1−*x*_Sb*_x_* was also prepared via TRMS and SPS [10]. Sub-micron grain (200–400 nm) structure was formed in SPSed bulks; thus, very low lattice thermal conductivity (*κ*_lat_ = *κ*_tot_ − *κ*_ele_, where *κ*_ele_ is the electronic thermal conductivity) ~2.4 W m^−1^ K^−1^ was obtained at 300 K due to the generation of highly dense grain boundary as well as the formation of point defects (Zr and Ti at Hf-site and Sb at Sn-site). 

In this study, we fabricated the polycrystalline bulks of Ti substituted Hf_0.5_Zr_0.5_NiSn_0.98_Sb_0.02_ half-Heusler compounds via a combined technique of TRMS and SPS and investigated the effect of Ti addition on the characteristics of phase evolution and TE transport properties. Despite the generation of a small amount of nano-scale secondary phases in melt-spun ribbons, single-phase half-Heusler alloys can be obtained after a short time of pressure-induced sintering by SPS, suggesting the formation of Hf-Zr-Ti and Sn-Sb point defects. We also found that the Ti addition suppressed the grain growth during the TRMS process; thus, smaller grain (~200 nm) structure compared to the pristine Hf_0.5_Zr_0.5_NiSn_0.98_Sb_0.02_ sample (~500 nm) was formed in SPSed (Hf_0.5_Zr_0.5_)_0.8_Ti_0.2_NiSn_0.98_Sb_0.02_. A peak *zT* of 0.47 was obtained at 800 K in Hf_0.4_Zr_0.4_Ti_0.2_NiSn_0.98_Sb_0.02,_ benefitting from reduced *κ*_lat_ due to the intensified point defect and grain boundary phonon scattering.

## 2. Materials and Methods

Polycrystalline bulks of (Hf_0.5_Zr_0.5_)_1−*x*_Ti*_x_*NiSn_0.98_Sb_0.02_ (*x* = 0, 0.1, 0.2, 0.3, and 0.4) were fabricated via arc melting, ball milling, and subsequent SPS. Compacted disc-type sample (~5 g mixture of Hf (Alfa Aesar, 99.6%), Zr (Alfa Aesar, 99.8%), Ti (Kojundo Chemical, 99.9%), Ni (Kojundo Chemical, 99.9%), Sn (Alfa Aesar, 99.85%), and 10 wt.% excess Sb (5N Plus, 99.999%) powders according to the designed composition) were used as starting materials for arc melting process under a high vacuum (<10^−5^ Torr). Ribbon-type samples (~1 mm in width, ~10 mm in length, and ~10 μm in thickness) were prepared from arc-melted ingot by using TRMS technique (Vacuum Rapid Solidification Process System, Y&I Tech, Paju, South Korea). The melt (by temperature-controlled induction heating) was injected by Ar pressure (~40 kPa) onto rotating Cu wheel (~250 mm in diameter). We set the Cu wheel rotating linear speed as 50 m s^−1^ since characteristic size of grains observed in melt-spun ribbons was converged to a minimum value at this condition. The acquired melt-spun ribbons were crushed into powders by using a high-energy ball mill (8000D, SPEX, New Jersey, USA) and sieved (<44 μm, 325 mesh). Then, powders were compacted into highly dense (>95% relative density) bulks (10 mm in diameter and 2 mm in thickness) by using SPS (SPS-5115, SPS Syntex, Tokyo, Japan) at 1103 K for 10 min under a uniaxial pressure of 60 MPa. 

The characteristic sizes of melt-spun ribbons (contact (where the ribbons make a direct contact to the Cu wheel, thus the solidification rate is maximized at contact surface) and free surfaces) and the grain sizes of SPSed bulks (fractured surface) were investigated by using scanning electron microscopy (SEM, JSM-7600F, JEOL, Tokyo, Japan). Phase formation behavior of Ti-substituted (Hf_0.5_Zr_0.5_)NiSn_0.98_Sb_0.02_ was confirmed by X-ray diffraction (XRD, Smartlab, Rigaku, Japan) with Cu K*α*1 radiation (*λ* = 1.5418 Å). Temperature-dependent *σ* and *S* values within temperature range of 300–800 K were measured by using a commercial measurement system (ZEM-3, ULVAC-RICO, Yokohama, Japan) under a He atmosphere. The *κ*_tot_ values were calculated by separate measurement of the sample density (*ρ*_s_), heat capacity (*C*_p_), and thermal diffusivity (*α*) according to equation of *κ*_tot_ = *ρ*_s_*C*_p_*α*. The *ρ*_s_ was measured at room temperature based on Archimedes’ principle. Additionally, the temperature dependences of *C*_p_ and *α* were obtained via analysis of differential scanning calorimetry (DSC, DSC8270, Rigaku, Tokyo, Japan) and laser-flash method (TC-1200RH, ULVAC-RICO, Yokohama, Japan), respectively.

## 3. Results and Discussions 

**Figure 1:**
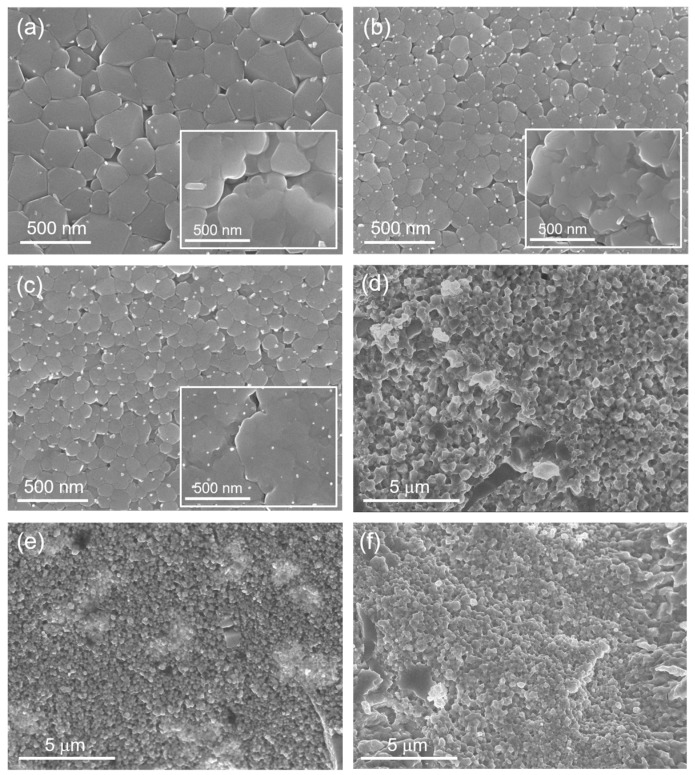
SEM (scanning electron microscopy) images for contact surfaces of melt-spun ribbons of (**a**) (Hf_0.5_Zr_0.5_)NiSn_0.98_Sb_0.02_, (**b**) (Hf_0__.5_Zr_0.5_)_0.8_Ti_0.2_NiSn_0.98_Sb_0.02_, and (**c**) (Hf_0__.5_Zr_0.5_)_0.7_Ti_0.3_NiSn_0.98_Sb_0.02_. Insets in (**a**–**c**) show the SEM images for free surfaces. SEM images for the fractured surfaces of SPS (spark plasma sintering) sintered bulks of (**d**) (Hf_0.5_Zr_0.5_)NiSn_0.98_Sb_0.02_, (**e**) (Hf_0__.5_Zr_0.5_)_0.8_Ti_0.2_NiSn_0.98_Sb_0.02_, and (**f**) (Hf_0__.5_Zr_0.5_)_0.7_Ti_0.3_NiSn_0.98_Sb_0.02_.

**Figure 2:**
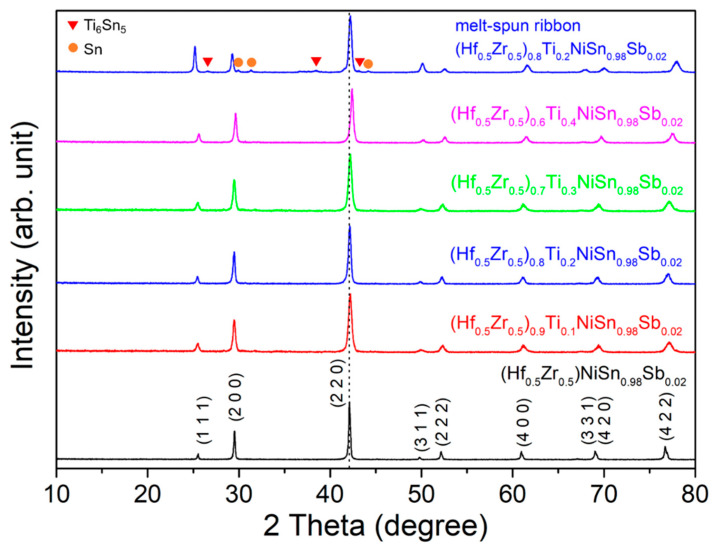
X-ray diffraction (XRD) patterns for the melt-spun ribbons of (Hf_0__.5_Zr_0.5_)_0.8_Ti_0.2_NiSn_0.98_Sb_0.02_ and sintered bulks of (Hf_0.5_Zr_0.5_)_1−*x*_Ti*_x_*NiSn_0.98_Sb_0.02_ (*x* = 0, 0.1, 0.2, 0.3, and 0.4).

Figure 1a–c show the SEM images of melt-spun ribbons (contact surface, insets show the SEM images for free surfaces) for (Hf_0.5_Zr_0.5_)NiSn_0.98_Sb_0.02_, (Hf_0__.5_Zr_0.5_)_0.8_Ti_0.2_NiSn_0.98_Sb_0.02_, and (Hf_0__.5_Zr_0.5_)_0.7_Ti_0.3_NiSn_0.98_Sb_0.02_, respectively. Sub-micron grain structure and dispersed white nanosized inclusions are clearly observed in all samples. In a previous study [8], we confirmed the compositions of nanoinclusions (binary Ti_6_Sn_5_ and elemental Sn). It is noted that the grain sizes observed in (a) and (b,c) show large differences; relatively larger grains (100–400 nm) were formed in the pristine (Hf_0.5_Zr_0.5_)NiSn_0.98_Sb_0.02_, while smaller and more uniform size grains (80–250 nm) were generated in the Ti-added samples ((Hf_0__.5_Zr_0.5_)_0.8_Ti_0.2_NiSn_0.98_Sb_0.02_ and (Hf_0__.5_Zr_0.5_)_0.7_Ti_0.3_NiSn_0.98_Sb_0.02_). This characteristic feature was also found in the SEM images for free surfaces of the ribbons (insets in Figure 1a–c), suggesting that the Ti might perturb the nucleation and growth of half-Heusler phase during the TRMS process due to the increased compositional complexity. We also carried out TRMS under higher Cu wheel rotating speed (60 m s^−1^) to reduce the *κ*_lat_ by decreasing grain size in melt-spun ribbons since the phonon mean free path of half-Heulser compounds such as TiNiSn_1−*x*_Sb*_x_* can be effectively decreased by decreasing the average grain diameter [11]. Reduced *κ*_lat_ by the intensified grain boundary phonon scattering have been theoretically and experimentally confirmed in previous studies [8,11,12]. However, the characteristic size observed in the contact surface was not significantly decreased. By using the melt-spinning technique with very high solidification rate ~10^6^ K s^−1^, nano-scale grains of TE materials, where the size of grain mainly depends on composition, can be obtained in other TE materials. Randomly shaped grains with the characteristic size of 20–80 nm were formed in the contact surface of melt-spun ribbons in skutterudite-based compounds (Co-Sb- and Fe-Co-Sb-based alloys) [13], while an amorphous phase was observed near the contact surface in the melt-spun ribbon of Bi_0.52_Sb_1.48_Te_3_, and nanocrystalline domains in the amorphous matrix and the 5–15 nm nanocrystalline regions were generated in a SPSed bulk sample [14]. Figure 1d–f show the SEM images for fractured surfaces of (Hf_0.5_Zr_0.5_)NiSn_0.98_Sb_0.02_, (Hf_0__.5_Zr_0.5_)_0.8_Ti_0.2_NiSn_0.98_Sb_0.02_, and (Hf_0__.5_Zr_0.5_)_0.7_Ti_0.3_NiSn_0.98_Sb_0.02_ bulk samples, representing that sub-micron grain structure in melt-spun ribbons was maintained even after SPS without severe grain growth during the sintering process. Due to the smaller grain size in melt-spun ribbon, SPSed bulk with much smaller grains (100–300 nm) compared to the pristine sample (200–800 nm) can be obtained in (Hf_0__.5_Zr_0.5_)_0.8_Ti_0.2_NiSn_0.98_Sb_0.02_ and (Hf_0__.5_Zr_0.5_)_0.7_Ti_0.3_NiSn_0.98_Sb_0.02_, which suggests that the density of grain boundary is increased by Ti addition. Almost the same features both in melt-spun ribbons and SPSed bulks were observed in all Ti-added samples. It is noted that the white-colored nanoinclusions observed both in the contact and free surfaces in the melt-spun ribbons (Figure 1a–c) are completely disappeared after sintering by SPS, which can also be confirmed by following XRD analysis shown in Figure 2.

To investigate the phase formation behavior and phase evolution during the SPS process, we carried out an XRD analysis for the SPSed bulks, represented in Figure 2. Peaks in all samples were indexed as a cubic F$\bar{4}$3m ZrNiSn phase, indicating that a single half-Heusler phase was fabricated and added Ti and Sb atoms were incorporated into Hf/Zr- and Sn-site. Secondary phases of Ti_6_Sn_5_ and Sn, generated in melt-spun ribbons, were diffused into the lattice during SPS due to the short diffusion distance between nano-scale phases as confirmed in SEM images for the fractured surfaces of SPSed bulks (Figure 1c,d). This phase evolution behavior during the SPS process was already demonstrated in the previous study on Ti_1−*x*_Hf*_x_*NiSn_1−*y*_Sb*_y_* half-Heusler alloys [8]. On the other hand, peak shift to higher 2 theta (~42.11° for (2 2 0) plane in (Hf_0.5_Zr_0.5_)NiSn_0.98_Sb_0.02_, ~42.21° in (Hf_0.5_Zr_0.5_)_0.9_Ti_0.1_NiSn_0.98_Sb_0.02_, ~42.19° in (Hf_0.5_Zr_0.5_)_0.8_Ti_0.2_NiSn_0.98_Sb_0.02_, ~42.23° in (Hf_0.5_Zr_0.5_)_0.7_Ti_0.3_NiSn_0.98_Sb_0.02_, and ~42.41° in (Hf_0.5_Zr_0.5_)_0.6_Ti_0.4_NiSn_0.98_Sb_0.02_) was observed in Ti-substituted samples, which means the decrease in lattice constant is another evidence for Ti substitution since the atomic radius of Ti (147 pm) is smaller than those of Hf (225 pm) and Zr (160 pm).

**Figure 3:**
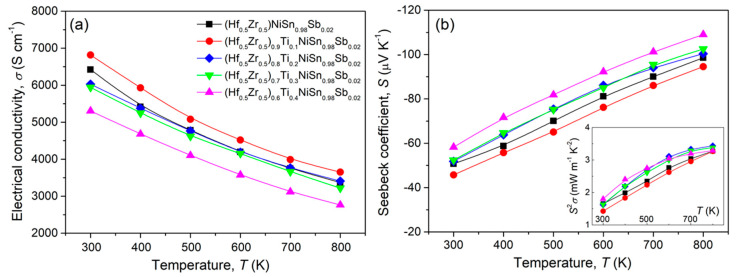
Temperature–dependent (**a**) electrical conductivity (*σ*) and (**b**) Seebeck coefficient (*S*) for the sintered bulks of (Hf_0.5_Zr_0.5_)_1−*x*_Ti*_x_*NiSn_0.98_Sb_0.02_ (*x* = 0, 0.1, 0.2, 0.3, and 0.4). Inset of (**b**) is the temperature dependence of power factor (*S*^2^*σ*).

Figure 3a shows the temperature-dependent *σ* of (Hf_0.5_Zr_0.5_)_1−*x*_Ti*_x_*NiSn_0.98_Sb_0.02_ (*x* = 0, 0.1, 0.2, 0.3, and 0.4) samples. Due to the carrier generation by Sb doping at Sn-site, all samples showed higher *σ* values over 5300 S cm^−1^ at 300 K (~200 S cm^−1^ at 300 K for Ti_1−*x*_Hf*_x_*NiSn [8]) and degenerate semiconducting behavior (decrease in *σ* with increasing temperature). Despite the same charge of Ti (Ti^4+^) with Hf (Hf^4+^) and Zr (Zr^4+^), variation in *σ* values according to Ti content was observed within the whole measured temperature range. As electron carriers are mainly generated by Sb doping at Sn-site, higher *σ* than that of the pristine (Hf_0.5_Zr_0.5_)NiSn_0.98_Sb_0.02_ obtained in (Hf_0.5_Zr_0.5_)_0.9_Ti_0.1_NiSn_0.98_Sb_0.02_ is considered to be related to a higher actual Sb doping content. This is due to the volatilization of Sb during the arc melting process. Additionally, the reason for the lower *σ* of (Hf_0.5_Zr_0.5_)_0.6_Ti_0.4_NiSn_0.98_Sb_0.02_ might be the decrease in mobility with increasing the density of point defect of substituted Ti. Figure 3b shows the measured *S* values as a function of temperature, which represents a typical trade-off relationship with *σ*. The lower absolute *S* values of (Hf_0.5_Zr_0.5_)_0.9_Ti_0.1_NiSn_0.98_Sb_0.02_ within the whole measured temperature range than those of the pristine (Hf_0.5_Zr_0.5_)NiSn_0.98_Sb_0.02_ are due to the increase in carrier concentration. A notable change in *S* value by Ti substitution was not detected; thus, resulting power factor (*S*^2^*σ*) values at 800 K were changed within a narrow range of 3.26–3.43 mW m^−1^ K^−2^ as shown in the inset of Figure 3b.

**Figure 4:**
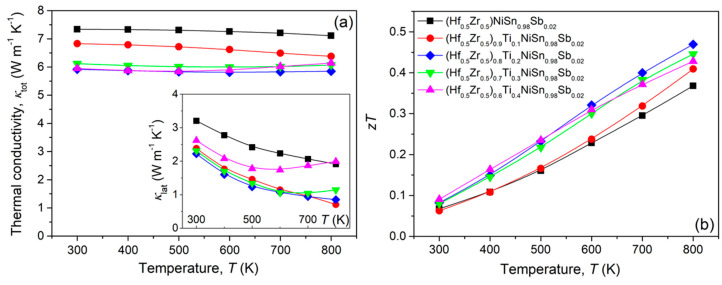
Temperature-dependent (**a**) total thermal conductivity (*κ*_tot_) and (**b**) dimensionless figure of merit (*zT*) for the sintered bulks of (Hf_0.5_Zr_0.5_)_1−*x*_Ti*_x_*NiSn_0.98_Sb_0.02_ (*x* = 0, 0.1, 0.2, 0.3, and 0.4). Inset of (**a**) is the temperature dependence of lattice thermal conductivity (*κ*_lat_).

Figure 4a shows the temperature-dependent *κ*_tot_ of (Hf_0.5_Zr_0.5_)_1−*x*_Ti*_x_*NiSn_0.98_Sb_0.02_ (*x* = 0, 0.1, 0.2, 0.3, and 0.4) samples. To investigate the change in phonon-scattering behavior by Ti addition, *κ*_lat_ values were estimated by calculation of *κ*_ele_ from the measured temperature-dependent *σ* (Figure 3a) based on Wiedemann–Franz law (*κ*_ele_ = *LσT*, where *L* is the Lorenz number). The *L* values as a function of temperature were obtained from the measured *S* (Figure 3b) by using Equation (3) with the assumption of a single parabolic and acoustic phonon scattering [15].

(3) $$L=1.5+\exp\left(-\frac{\left|S\right|}{116}\right)$$

In Equation (3), *L* is in 10^−8^ W Ω K^−2^ and *S* in μV K^−1^. Calculated *L* values were ranged between 2.105 and 2.174 × 10^−8^ W Ω K^−2^ at 300 K and 1.891–1.943 × 10^−8^ W Ω K^−2^ at 800 K, respectively. The inset of Figure 4a shows the estimated *κ*_lat_ as a function of temperature.

The *κ*_lat_ values in all samples were decreased with increasing temperature except for (Hf_0__.5_Zr_0.5_)_0.6_Ti_0.4_NiSn_0.98_Sb_0.02_. An increase in *κ*_lat_ observed in (Hf_0__.5_Zr_0.5_)_0.6_Ti_0.4_NiSn_0.98_Sb_0.02_ at >600 K is considered to be related to the contribution from bipolar conduction. It should be noted that the *κ*_lat_ values of the pristine (Hf_0.5_Zr_0.5_)NiSn_0.98_Sb_0.02_ sample (~3.21 W m^−1^ K^−1^ at 300 K and ~1.92 W m^−1^ K^−1^ at 800 K) were significantly reduced by Ti addition (~2.05 W m^−1^ K^−1^ at 300 K and ~0.61 W m^−1^ K^−1^ at 800 K in (Hf_0__.5_Zr_0.5_)_0.8_Ti_0.2_NiSn_0.98_Sb_0.02_)) due to the synergetic effect of the intensified phonon scattering from highly dense point defects (Ti at Hf/Zr-site and Sb at Sn-site) and grain boundaries. The large mass difference between Ti (47.867 u) and Hf (178.49 u)/Zr (91.224 u) might trigger the phonon scattering by mass fluctuation in Ti-substituted samples since the phonon scattering parameter by point defect is related to the rate of change of the atomic mass (Δ*M*/*M*) [16]. Additionally, grain boundary can effectively reduce the phonon mean free path at low frequency due to the relaxation time associated with grain boundary scattering of *τ*_GB_^−1^ = *v*/*d* (where *v* is the average phonon velocity and *d* is the grain size) [17]. 

The *zT* values of the SPSed bulks of (Hf_0.5_Zr_0.5_)_1−*x*_Ti*_x_*NiSn_0.98_Sb_0.02_ (*x* = 0, 0.1, 0.2, 0.3, and 0.4) samples are shown in Figure 4b. A peak *zT* of 0.47 was observed in (Hf_0.5_Zr_0.5_)_0.8_Ti_0.2_NiSn_0.98_Sb_0.02_ at 800 K due to simultaneously obtained high power factor and low *κ*_lat_. Compared to the previous results reported in *n*-type HfNiSn-based, half-Heusler alloys [5], *zT* values in this study are moderate. However, acquired results and related discussions provide meaningful compositional tuning approaches connected with processing technologies. 

## 4. Conclusions

Polycrystalline bulks of submicron-grained Hf_0.5_Zr_0.5_NiSn_0.98_Sb_0.02_–based, half-Heusler alloys were fabricated by using temperature-regulated melt-spinning and spark plasma sintering. We found that the phase evolution behavior during the melt spinning process can be controlled by Ti addition, resulting in the suppression of grain growth due to compositional complexity. Due to the increased density of multi-dimensional defect structures, including 0-dimensional point defects (Ti at Hf/Zr-site and Sb at Sn-site) and two-dimensional grain boundaries, very low lattice thermal conductivity values of ~2.05 W m^−1^ K^−1^ at 300 K and ~0.61 W m^−1^ K^−1^ at 800 K and a peak *zT* of 0.47 at 800 K were obtained in (Hf_0__.5_Zr_0.5_)_0.8_Ti_0.2_NiSn_0.98_Sb_0.02_.

## Data Availability

Data sharng is not applicable for this article.
